# SELVa: Simulator of evolution with landscape variation

**DOI:** 10.1371/journal.pone.0242225

**Published:** 2020-12-02

**Authors:** Elena Nabieva, Georgii A. Bazykin

**Affiliations:** 1 Skolkovo Institute of Science and Technology, Moscow, Russia; 2 Kharkevich Institute of Information Transmission Problems, Moscow, Russia; University of Pittsburgh, UNITED STATES

## Abstract

Organisms evolve to increase their fitness, a process that may be described as climbing the fitness landscape. However, the fitness landscape of an individual site, i.e., the vector of fitness values corresponding to different variants at this site, can itself change with time due to changes in the environment or substitutions at other epistatically interacting sites. While there exist a number of simulators for modeling different aspects of molecular evolution, very few can accommodate changing landscapes. We present SELVa, the Simulator of Evolution with Landscape Variation, aimed at modeling the substitution process under a changing single-position fitness landscape in a set of evolving lineages that form a phylogeny of arbitrary shape. Written in Java and distributed as an executable jar file, SELVa provides a flexible framework that allows the user to choose from a number of implemented rules governing landscape change.

## Introduction

The differences between species arise in the course of evolution due to substitutions, that is, mutations that spread to fixation in evolving lineages. The properties of the substitution process are shaped by mutation giving rise to new variants and by selection favoring some variants over others. Computer simulation methods have established themselves as invaluable tools to infer the characteristics of these processes (for review, see [1]; for a catalogue, see [2]). Among the diversity of evolutionary simulators, one family of programs models evolution along a given phylogeny. Over the years, simulation methods in this category have grown increasingly sophisticated in the models that they implement: from only evolving nucleotide, codon or amino acid sequences by substitutions governed by one of several well-established models [3,4], to allowing insertions and deletions [5,6], to modeling evolution under domain preservation or structural constraints [7], and further on. These programs generally permit the user to model inhomogeneity of the evolutionary process among different sites; they may do so, for example, by designating certain sites or groups of sites as invariable or, conversely, as having increased or decreased rates of evolution.

An important feature of evolution is that its parameters themselves change with time and/or along different phylogenetic branches. In particular, fitness landscapes change over time, whether due to changing external pressures, such as environmental changes or pathogen-host interactions, or, in the case of single-position fitness landscapes, because of epistatic interactions with other sites that are also evolving [8,9]. For example, a study in *Drosophila* found that fitness fluctuates at rates comparable to those of nucleotide changes [8]. Understanding the effects of landscape change can lead to insights into forces governing molecular evolution [10]. Studying these effects, therefore, calls for an evolutionary simulator capable of modeling landscape changes. Some existing simulators do allow model parameters to vary among branches [3,5,6,11,12] or at a specified time on a branch [13], yet these capabilities are rather limited. While a recently published individual-based forward simulator of population dynamics SANTA-SIM [14] accommodates a number of scenarios for changing selection, it does not permit simulation of evolution along a prespecified phylogeny. We therefore developed SELVa, the Simulator of Evolution with Landscape Variation. SELVa focuses on the *single-position* functional landscape and provides a range of settings that allow the user to parametrize landscape change and specify when, where, and how the landscape change occurs.

## Results and discussion

### Usage

SELVa is run from the command line. The user provides the program with a rooted phylogenetic tree in Newick format and a text configuration file detailing the options of the simulation. The core of the configuration specifies the rules for evolving one or more fitness landscapes. For each landscape, the user may choose to have multiple positions governed by it, which are then viewed as making up a sequence evolving under a shared landscape. It is possible to have the landscapes governed by completely different rules, or to have different landscapes generated from the same set of rules (distributions), as is described below in the “Parallel simulations” paragraph. Each landscape is defined by a vector of population size-scaled additive fitness values [15] for each allele. This vector is henceforth referred to as just the “fitness vector”. The simulation starts with an initial fitness landscape (vector) and either supplied by the user in a separate text file or generated from one of the supported probability distributions (currently, they include the gamma and the lognormal) with user-specified parameters. The user may also provide a mutation-rate matrix that remains fixed throughout the course of the simulation; if not provided, all of its entries are assumed to be 1. At the start of the simulation, SELVa generates the root state for each position by sampling from the stationary distribution obtained from the initial fitness vector and the mutation rate matrix [16]. The user also has the option of supplying the root sequence explicitly; it is up to the user to ensure the degree of likelihood of that sequence under the initial stationary distribution. In the course of a simulation, the landscape may change according to the user-provided rules, as described below.

Upon completion, SELVa prints out the sequence(s) generated at each node of the phylogenetic tree in the course of the simulation, and, optionally, the times of landscape changes and the fitness vectors generated at those times, providing the user with detailed information about the course of the simulation.

### Landscape change timing

**Fig 1:**
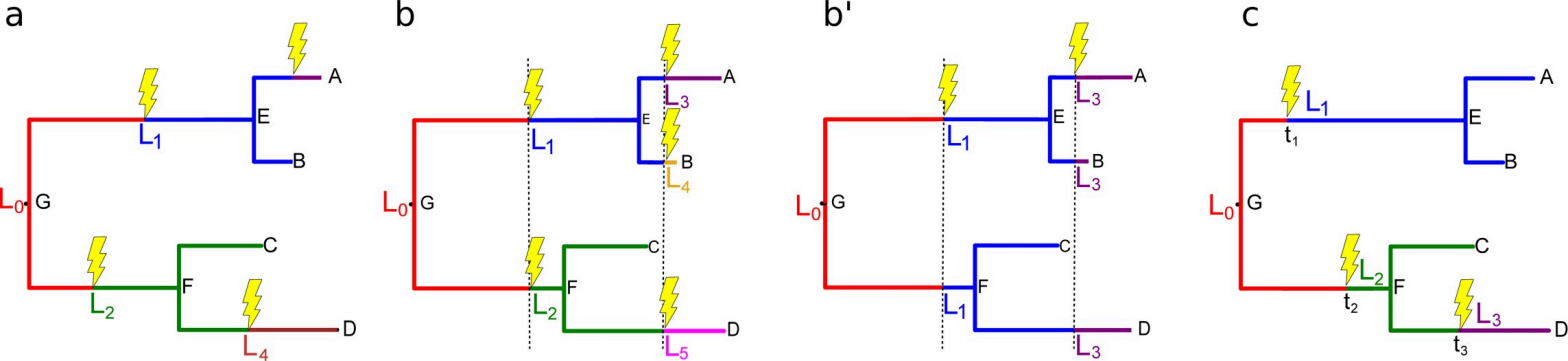
Landscape change options. The landscape change regimes currently supported by SELVa. In all scenarios, the simulation begins at the root node G with the landscape L_0_. A, B, C, D are leaf nodes (“extant sequences”), and E and F are internal nodes. “Lightning strikes” denote landscape change events; colors and L_i_ labels correspond to landscapes that govern evolution along the corresponding subtree. (a) The landscape change occurs stochastically: on the branches leading to nodes E, F, A, and D. (b and b’) The landscape change occurs at evenly spaced time intervals (denoted by dashed vertical lines), and the new fitness landscapes are independent for different branches (b) or shared among parallel branches (b’), as reflected in the landscape labels and subtree colors. (c) The landscape change occurs at user-specified branch coordinates t_1_, t_2_, t_3_; with this option, the user may specify the exact fitness vectors describing L_1_, L_2_, and L_3_, or generate these landscapes probabilistically as with the other settings.

SELVa allows for several regimes governing the timing of landscape changes. They can occur stochastically as a Poisson process (Fig 1A), or deterministically, either at evenly spaced time intervals (Fig 1B and 1B’) or at user-specified positions in the tree (Fig 1C). In the deterministic evenly-spaced case, the landscape values can be independent between branches (Fig 1B) or shared by parallel branches (Fig 1B’). The user additionally has the option of providing the exact times (“branch coordinates”) of landscape change (Fig 1C) and, if desired, the new fitness values to change to at those times.

### Generation of the new landscapes

The values of the new fitness vector can be independently sampled from the same distribution as the initial fitness vector; they can be obtained from the previous fitness vector by randomly permuting its values; they can be explicitly specified by the user (currently, only if the landscape change times are also manually specified by the user); or they can be derived from the previous fitness vector by either increasing or decreasing the fitness of the allele currently occupying the site. The last option permits the modeling of processes in which the fitness of the current allele increases or decreases with time, e.g., as might happen as a result of epistatic interactions with other sites [17]).

### Parallel simulations

SELVa can carry out multiple parallel independent landscapes in a single execution, amounting to multiple independent simulations carried out in parallel. Here, there are two possible scenarios. One is simulating multiple landscapes from the same set of rules (same distribution and parameters, but different instantiation history). This option is suited, for example, to studying the effects of landscape change by aggregating the results of a large number of simulations governed by the same parameters. The other option is having completely unrelated landscapes that govern different subsequences. This latter case is more suitable to simulating situations where certain parts of a sequence are subject to different selection. If multiple processors are available, these parallel simulations can be divided among multiple threads.

The full options and their details are given in the Manual (available at https://github.com/bazykinlab/SELVa/blob/master/SELVa_manual.pdf).

#### Potential applications

SELVa is designed for modeling scenarios in which the single-position fitness landscape changes with time. Some examples of such scenarios are:

- A viral sequence is evolving under pressure from the immune system, which causes the current allele to become progressively less fit as the immune system adapts to it. This is modeled by decreasing the fitness of the current allele at regular time intervals. The length of the time interval can be adjusted for a resolution vs. speed tradeoff.
- The current allele in a certain sequence position is gaining fitness due to epistatic interactions with other alleles. Although SELVa does not model such interactions explicitly, this regime can be used when epistasis is believed to increase the fitness of the existing allele with time. In particular, it is modeled by increasing the fitness of the current allele at regular time intervals.
- The fitness of the current allele changes at random time points corresponding to stochastic changes in the environment. This is modeled by choosing the stochastic landscape change option and resampling the new fitness vector from the same distribution.

#### Validation

**Fig 2:**
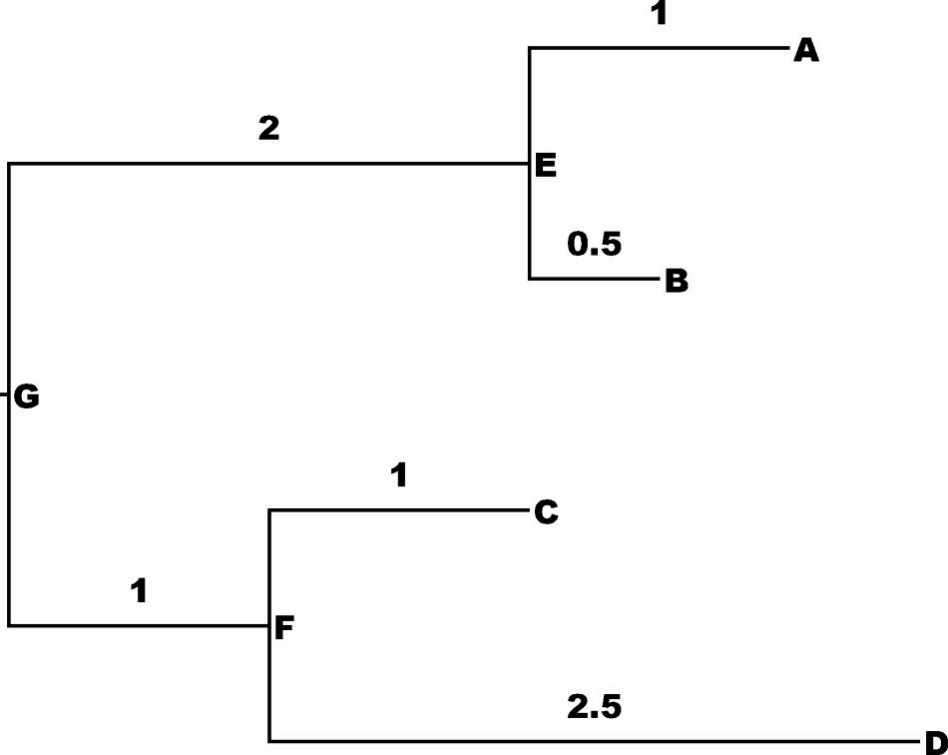
The “Simple” tree.

We performed a panel of tests on a number of trees. First, we evaluated whether the stochastic landscape change model conforms to expected behavior. To do so, we ran the stochastic landscape change model with different choices of the Poisson rate *λ* that governs the rate of landscape change. Under the Poisson model, we expect the number of landscape changes to be close to λ*(sum of branch lengths). The fitness values were selected from a lognormal distribution with μ = 0, σ = 0.5, with a new landscape selected by shuffling the fitness values of the vector. We performed these tests on a “Simple” manually created tree with four leaves and sum of branch lengths equal to 8 (Fig 2); on a tree of 100 *Saccaromyces* mtDNA sequences, sum of branch lengths 0.1585 ([18], TreeBase [19] entry 87814); on a tree obtained by simulating a birth-death process on 100 species with rate 1 (sum of branch lengths 5622.5); and on a metazoan mitochondrial tree of 3558 species, sum of branch lengths: 95.0 (Galina Klink, personal communication). The trees are provided in Newick format in S1 File.

The mean and standard deviation of the number of landscape changes on these trees with different value of the landscape change rate *λ* are given in Table 1.

**Table 1:** The mean and standard deviation (in parentheses) of the number of landscape changes on the given tree under the stochastic model with the Poisson parameter *λ*.

*Tree*	***“Simple”***		***Saccharomyces mtDNA***		***Simulated birth-death***		***Metazoan mtDNA***	
*#leaves*:	*4*		*100*		*100*		*3558*	
*sum lengths*:	*8*		*0*.*1585*		*5622*.*5*		*95*.*0*	
	*expected*	*observed*	*expected*	*observed*	*expected*	*observed*	*expected*	*observed*
*λ* *= 0*.*1*	*0*.*8*	*0*.*8 (0*.*920)*	*0*.*01585*	*0*.*0183 (0*.*135)*	*5562*.*25*	*562*.*6 (24*.*0)*	*9*.*5*	*9*.*69 (3*.*61)*
*λ* *= 1*	*8*	*7*.*909 (2*.*83)*	*0*.*1585*	*0*.*152 (0*.*404)*	*5622*.*5*	*5623*.*956 (78*.*28)*	*95*	*95*.*14 (9*.*69)*
*λ* *= 10*	*80*	*79*.*96 (8*.*891)*	*1*.*585*	*1*.*583 (1*.*23)*	*56226*	*56237*.*02 (208*.0)*	*950*	*950*.*16 (33*.*0)*

*The simulator ran out of memory on 1000 parallel instances on the birth-death tree with *λ* *= 10*, so the result is shown for 100 instances.

We then tested the deterministic change option on the “Simple” tree where the landscape change times can be examined manually. An instance of the output is given in S2 File. The changes took place as expected, although floating-point effects led to minor inconsistencies, such as a landscape change just before node D, which would not have had time to take effect.

In order to test the setting in which the fitness of the current allele increases (or decreases) with time, we ran the simulator on a “comb-like” tree (every inner node has two children, one of which is a leaf) of depth 100 with branch length 1 (S1 File). For each node, we counted the number of sites that changed between the node and its parents. As expected, we observed that the fraction of sites where substitution occurs between adjacent nodes drops as a function of time, as the current allele becomes more fit and therefore less likely to be changed. Meanwhile, in a no-change flat-landscape setting, the fraction of sites that get substituted remains constant at about 50% (S1 Fig in S2 File).

Finally, we checked that the allele distribution converges to the new landscape’s stationary distribution following a landscape change and indeed find it to be the case (S2 Fig in S2 File).

For a close-up look at what happens to the allele distribution following a landscape change, we looked at the “Simple” tree and set a random landscape change at 0.5 before node F. We observe that, as expected, the allele distribution at F has not yet converged to the stationary distribution, while nodes further down that subtree already contain alleles at frequencies that are very similar to the stationary one (S2 File).

#### Performance

We evaluated the performance of SELVa on a more challenging mitochondrial tree of 3558 species (S1 Fig in S2 File). The height of the tree (longest path from root to leaf) is 0.97 substitutions per site, and the sum of branch lengths is 95.02. The initial (population size-) scaled fitness landscape was set to be (1, 0.1, 0.1, …, 0.1). The transition rate matrix was normalized, so that one substitution was expected to take place per site per unit time. New fitness vectors were generated by randomly permuting the values of the previous fitness vector. The following landscape change regimes were tested: no change, stochastic change with rate 1.0 (per branch length unit), stochastic change with rate 10.0. A single simulation for a sequence of length 1, a single simulation for a sequence of length 1000, and 1000 parallel simulations for sequences of length 1 were tested. To evaluate the effect of keeping track of and printing out landscape change times and intermediate landscape values, we tested the performance with and without that option. We averaged the results over 100 independent runs of SELVa (which, for the 1000 parallel simulations case, means 100 000 different actual simulations).

The simulations were run on a Samsung Notebook 9 with Intel Core i7-6500U 2.50GHz processor and 8 GB total RAM. Multi-simulation runs were not parallelized.

The results are summarized in S1 Table in S2 File.

The infrastructure associated with each simulation run is the most computationally expensive aspect of the program, with the infrastructure for keeping track of the landscape information adding some cost to it. λ is the instantaneous rate of landscape change.

## Implementation

SELVa is implemented in Java and distributed as an executable jar file. It requires installation of no additional packages.

### Simulation details

SELVa employs the widely used Gillespie algorithm [20], in particular, the general simulation framework of Chapter 12.6.1.3 of [16]. We provide the details of this framework and the specifics of its implementation below. SELVa simulates molecular evolution as an event-driven forward-in-time process of substitution in a sequence of a specified length, with positions corresponding to nucleotides, amino acids, or indeed any type of sequence variants. A fitness landscape is represented by a vector of (population size-) scaled additive fitness value for each character in the sequence alphabet (for example, amino acid). The user may also provide a mutation rate matrix *M* that remains fixed throughout the course of the simulation; if not provided, all of its entries are assumed to be 1. The fitness vector (and the mutation rate matrix) are used to derive the substitution rate matrix *Q = {q*_*ij*_*}* that contains the instantaneous rates of substitution from allele *i* to allele *j*, and the stationary distribution vector π for the current landscape [15]. Specifically, the scaled fitness vector (*F*_*i*_ *= 2Nf*_*i*_) is first converted to the unnormalized substitution rate matrix Q^raw^, with $q_{ij,i\neq j}^{raw}=M_{ij}\frac{F_{j}-F_{i}}{1-e^{F_{i}-F_{j}}}$ proportional to the instantaneous rate of substitution of allele *i* to allele *j*, and $q_{i}^{raw}=q_{ii}^{raw}=-\sum_{j:j\neq i}q_{ij,\;i\neq j}^{raw}$ the negative of the total rate of substitution *from* allele *i*. If *F*_*i*_ *= F*_*j*_, *q*_*ij*_^*raw*^ is set to *M*_*ij*_. The corresponding stationary distribution vector π is then computed by solving the system of equations $\pi Q=0;\;\Sigma_{i}\pi_{i}=1$ [16]; alternatively, if the mutation rate matrix is not provided (i.e., all entries are considered to be equal to 1), *ν* is calculated as $\pi_{i}=\frac{exp\;\left(F_{i}\right)}{\sum_{j}exp\;\left(F_{j}\right)}$ [15]. Then, the expected substitution rate for a position *i* is $-\sum_{i}q_{i}^{raw}\pi_{i}.$Dividing Q^raw^ by produces Q^normalized^, such that $-\sum_{i}q_{i}^{normalized}\pi_{i}=1$, i.e., the expected substitution rate is equal to 1, that is, one substitution is expected to occur per site per unit of evolutionary branch length. Following the practice of evolver [3], normalization is the default behavior. Alternatively, we can choose to scale Q^raw^ to get the expected substitution rate to be 1 only for the flat fitness vector such as [1,…,1] (all substitutions are neutral) whose corresponding Q^raw^ consists of 1’s off the diagonal and–(|A|-1) on the diagonal, where |A| is the alphabet size. Q^scaled^ is then obtained by dividing Q^raw^ by $\sum_{\left|A\right|}\frac{1}{\left|A\right|}\left(\left|A\right|-1\right)=\left(\left|A\right|-1\right),\frac{1}{\left|A\right|}$ being is the stationary probability of any allele for the flat fitness vector. If the matrix is left unnormalized, the user has the option of scaling the stochastic landscape change rate to it or not. We expect very few users to forego Q normalization.

The substitution process for a single site currently occupied by allele *i* is modeled as a Markov chain with the expected inter-event time *1/-q*_*i*_, where $-q_{i}=\sum_{j\neq i}q_{ij}$ is the total rate of transition away from *i*. For longer sequences, the transition rates for all sites are summed to obtain the rate of a substitution event occurring at *any* site, and then the site where a substitution occurs is chosen at random proportionally to the rate of transition away from the allele occupying it. Once a site occupied by an allele *i* is chosen to be mutated, its new allele *j* is selected proportionally to *q*_*ij*_. This process is repeated along each branch, starting at the root of the tree; after a substitution occurs, the waiting time until the next substitution is adjusted to reflect the new allele. If the waiting time until the next event exceeds the remaining branch length, no substitution occurs on the remainder of the branch.

We adapt this widely-used [6,7,13,21] framework to accommodate stochastically or deterministically scheduled landscape changes. In the stochastic case, the landscape change occurs as a Poisson process; in the deterministic case, it occurs at evenly-spaced intervals, or at user-specified positions on the tree.

In the stochastic case, we add to the set of possible stochastic events (i.e., substitutions at the sequence sites) a landscape-change event governed by the user-specified rate. The calculation of the inter-event waiting time and the choice of the next event are then carried out as described above by treating the landscape change event as an extra “site” whose transition rate is set by the user. The landscape change event causes a recalculation of the matrix Q and the vector π, and the subsequent substitution process is governed by their new values.

For deterministic landscape change timing, two processes are simulated simultaneously: the stochastic sequence substitutions, carried out as described above, and the deterministically-scheduled landscape change. At each step of the simulation, SELVa generates a waiting time until the next Poisson (i.e., substitution) event. Then, this waiting time is compared to the time remaining until the next deterministic landscape change. If the next event is the stochastic substitution event, then it takes place as described above, and the time remaining until the end of the branch or until the next deterministically-scheduled landscape change is adjusted. If the next event is a deterministically-scheduled landscape change, then the change is carried out, and a new stochastic waiting time for the next substitution event is generated using the new landscape. If a branch splits into two children before the next deterministic event, that event is scheduled to occur on both daughter branches.

The simulations are implemented using the Breadth-First Search algorithm on the provided phylogenetic tree.

## Conclusion

SELVa is a novel evolutionary simulator focused specifically on modeling the effect of changes in the fitness landscapes. By providing the user with a variety of options for specifying the regime and the specifics of fitness landscape changes, it fills a hitherto vacant need for exploring the effect of single-position fitness landscapes dynamics on the substitution process along a phylogeny.

## Supporting information

S1 File (TXT)

S2 File (PDF)
