# Supplementary material for: SELVa: Simulator of evolution with landscape variation
Source: PLoS One. 2020 Dec 2;15(12):e0242225. doi: 10.1371/journal.pone.0242225 (PMC7710038; doi:10.1371/journal.pone.0242225)
Supplement: S2 File — (PDF) [file pone.0242225.s002.pdf]

S2 File. Supplementary Results

S2. Times of landscape change under one run of the deterministic mode on the "Simple" tree.

We ran deterministic landscape change on the Simple tree with the fixed number of intervals (6) and the fixed interval length (0.5) settings. As the maximal leaf depth in the tree is 3.5, these two settings should result in the same landscape change times. We report the actual output below.

Landscape changes are encoded in FASTA-like format as branch times remaining until the node in the "header". Note the discrepancies that are due to floating-point issues.

Deterministic landscape change, fixed number of intervals: 6
>A
[1.0, 0.5];
>B
[0.5];
>C
[1.0, 0.5];
>D
[2.5, 2.0, 1.5, 0.9999999999999999, 0.4999999999999999];
>E
[1.5, 1.0, 0.5];
>F
[0.5];
>G
[0.0];

Deterministic landscape change,, interval length = 0.5:
>A
[1.0, 0.5];
>B
[0.5];
>C
[1.0, 0.5];
>D
[2.5, 2.0000000000000004, 1.5000000000000002, 1.0000000000000002, 0.5000000000000002, 2.220446049250313E-16];
>E
[1.5, 1.0, 0.5];
>F
[0.5];
>G
[0.0];

Fig S1. The percentage of sites (of a 100-character sequence) that change between child and parent on a "comb-like" graph as a function of tree distance, starting at a flat fitness landscape (1,1,1,...). The fitness is increased by 2 every 10 tree steps (hence the "jumps" in the plot at every 10 tree steps). For comparison, the corresponding distribution with no change from the flat landscape is given.

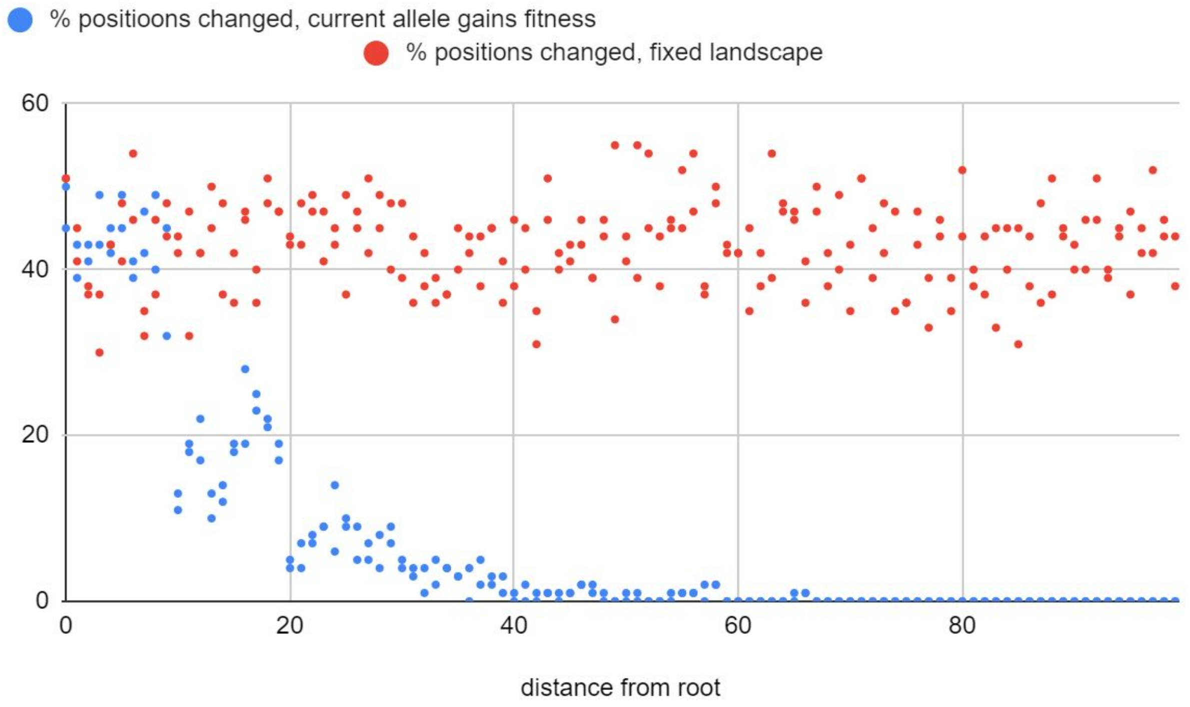

S3. Convergence of allele distribution

For this test, we chose a four-letter alphabet (ACGT). The simulation was run on the "Simple" tree and the letters correspond to the node labels. In each case, the length of the sequence was 1000, so the distribution of alleles was obtained by averaging over 1000 characters that were governed by a single landscape.

In the first setting, we ran the stochastic landscape change with rate 1 on 1000 independent landscapes, which resulted in us sampling a variety of distances from a landscape change to a node. The initial landscape was sampled from a lognormal distribution with σ = 0.5, and subsequent landscapes were obtained by randomly shuffling the previous one. For each node, we plotted the Kullback-Liebler divergence between the observed allele distribution and the stationary distribution corresponding to that node's landscape.

Fig S2. The Kullback-Liebler divergence between the observed allele distribution and the stationary distribution for the current landscape, as a function of time since landscape change.

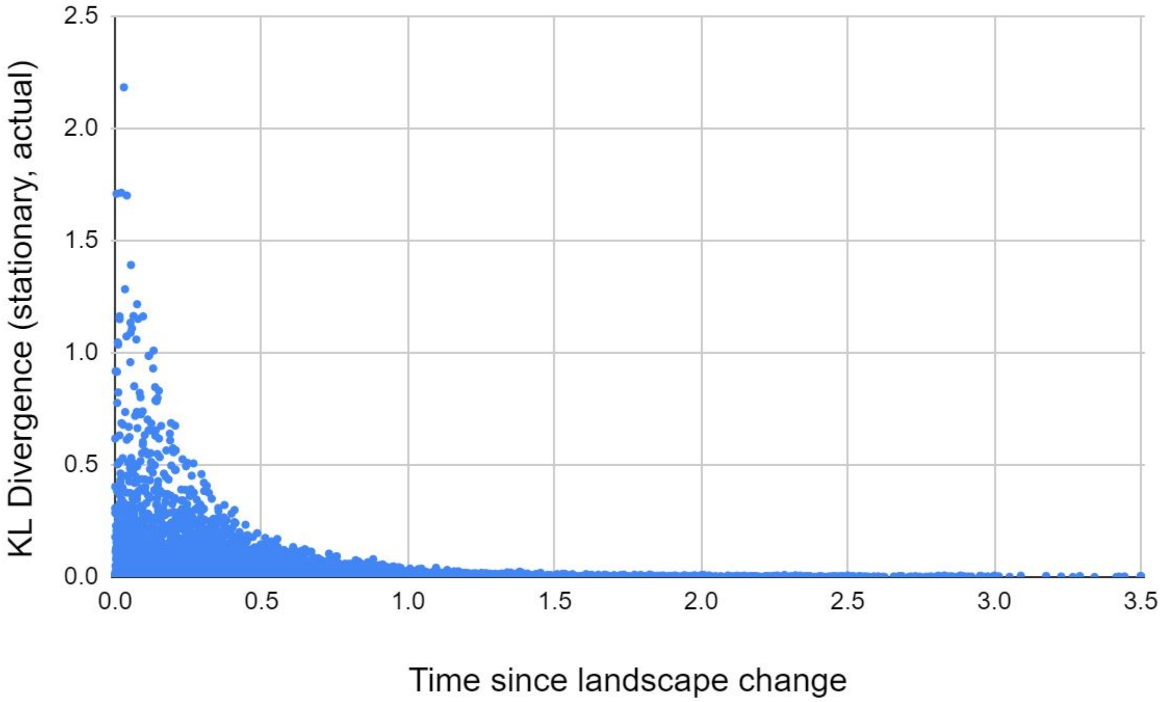

We then looked at what happens to the allele distribution for a single instance with a single change. In this setting, the fitness values were sampled from a lognormal distribution with $\sigma = 2$. The landscape was changed at F-0.5; the change thus affected nodes F, C, D, whereas the remaining nodes were subject to the root landscape.

In this run of the simulator, the following fitness landscapes were obtained:

Initial fitness vector (at *G*):
[0.3812915700127736, 0.1631077359253145, 2.5978647185823713, 0.011284116893212468]

The corresponding stationary distribution:
[0.08568584151364686, 0.0688894997492795, 0.7862389474738725, 0.059185711263201116]

The new fitness vector (at *F*-0.5):
[9.554927874193854, 0.047984806770162716, 7.995508363633427, 0.07265113093487115]

The corresponding stationary distribution:
[0.8261672873134266, 6.141226012893414E-5, 0.17370835451458297, 6.294591186148217E-5]

The following allele distributions were obtained over 1000-letter sequences sharing these landscapes. We copy the stationary distributions π for reference.

	A	C	G	T
π at root:	0.0857	0.0689	0.7862	0.0592
G	0.0856	0.0679	0.7885	0.0580
A	0.0870	0.0709	0.7852	0.0569
E	0.0902	0.0686	0.7794	0.0618
B	0.0892	0.0676	0.7819	0.0613
π at F-0.5	0.8262	0.0001	0.1737	0.0001
C	0.8306	0.0002	0.1692	0.0000
F	0.7054	0.0002	0.2944	0.0000
D	0.8229	0.0000	0.1771	0.0000

Supplementary Table 1: average running times and memory consumption for stochastic landscape change.

seq length	# parallel simul.	print landscapes?	no change		stochastic λ = 1		stochastic λ = 10	
1	1	yes	0.35	28.8	0.42	30.1	0.45	30.2
		no	0.32	25.6	0.34	26.3	0.36	32.7
1000	1	yes	1.25	42.9	1.6	43.6	1.37	52.2
		no	1.24	43	1.3	41.9	1.27	44.7
1	1000	yes	9.5	776.7	14.9	840.7	28.33	1070.1
		no	1.21	40.4	9.3	745	18.2	889.8
			**time (sec)**	**memory (MB)**	**time (sec)**	**memory (MB)**	**time (sec)**	**memory (MB)**
